# Impact of Digital Workflow Integration on Fixed Prosthodontics: A Review of Advances and Clinical Outcomes

**DOI:** 10.7759/cureus.72286

**Published:** 2024-10-24

**Authors:** Layla I Abdulkarim, Faisal Saad S Alharamlah, Razan M Abubshait, Deemah A Alotaibi, Anas O Abouonq

**Affiliations:** 1 Dentistry, College of Dentistry, Imam Abdulrahman Bin Faisal University, Dammam, SAU; 2 Dentistry, College of Dental Medicine, University of Sharjah, Khobar, SAU; 3 Prosthodontics, Aljouf University, Khobar, SAU

**Keywords:** 3d printing, accuracy, cad/cam, clinical outcomes, digital workflows, intraoral scanners, patient satisfaction, prosthetic restorations, prosthodontics, time efficiency

## Abstract

The field of prosthodontics has seen substantial advancements with the integration of digital workflows, which have revolutionized traditional clinical practices. These digital technologies, including intraoral scanners (IOS), computer-aided design (CAD), and computer-aided manufacturing (CAM), have enhanced the precision, efficiency, and overall quality of prosthetic restorations. This review explores the clinical outcomes of adopting digital workflows in prosthodontics, with a focus on their impact on accuracy, time efficiency, and patient satisfaction. Additionally, the review addresses the challenges and barriers to widespread adoption, such as high equipment costs and the need for continuous updates and training. A comprehensive literature review was conducted, selecting peer-reviewed studies that focused on the use of digital workflows in tooth-borne and implant-supported prostheses. Findings suggest that digital workflows provide superior accuracy in prosthesis fabrication and significantly reduce treatment time, particularly with the advent of chairside CAD/CAM systems that enable same-day restorations. Furthermore, patient satisfaction is improved due to increased comfort and reduced treatment duration. However, barriers such as the financial investment and learning curve remain obstacles to broader implementation. The review highlights the transformative potential of digital workflows in prosthodontics and emphasizes the need for more long-term clinical studies to further establish their efficacy.

## Introduction and background

The field of prosthodontics has undergone a significant transformation with the advent of digital technologies, which have reshaped clinical workflows and introduced new avenues for improving patient care. While traditional methods heavily rely on manual techniques and physical impressions, digital workflows offer alternatives that focus on enhanced precision, patient comfort, and streamlined processes. Digital tools such as intraoral scanners (IOS), computer-aided design (CAD), and computer-aided manufacturing (CAM) systems allow for the fabrication of dental prostheses through fully digitized processes. These technologies have not only optimized the design and production of restorations but have also enhanced the accuracy of implant placement and prosthetic fit, which is critical in both tooth-borne and implant-supported restorations [1,2].

One key advantage of digital workflows is their potential to enhance the accuracy and speed of prosthesis fabrication. Intraoral scanners, for instance, capture detailed 3D images of the dental arch, mitigating some of the errors associated with traditional impression materials [3]. This precise data collection enables the creation of highly accurate prostheses that can be designed using CAD software and transferred to CAM systems for milling or 3D printing using materials like zirconia, lithium disilicate, or hybrid ceramics [4,5]. However, it is essential to note that while chairside time is often reduced, more time may be required during the planning phase to ensure optimal results.

The increasing use of digital workflows also offers the potential for more efficient production processes, which can reduce the time required to create prosthetic restorations. However, the improvement in patient outcomes remains somewhat subjective, as expert practitioners using conventional methods can still achieve equally or, in some cases, better results. Digital impressions, while faster and more comfortable for patients, primarily enhance communication between the dentist, laboratory, and patient, allowing for greater customization and predictability in the final outcome [1].

Despite these advantages, challenges remain in integrating digital workflows in prosthodontics. The initial investment in digital equipment, the learning curve associated with adopting new technologies, and the need for regular software and hardware updates are significant barriers to widespread adoption. Furthermore, while short-term benefits are well documented, long-term clinical studies are still necessary to establish the efficacy of digital workflows in complex cases, particularly in multi-unit restorations [6,7].

In conclusion, the digitalization of prosthodontic workflows offers numerous advantages, including precision, efficiency, and enhanced customization. However, these benefits should be weighed against the increased planning time and subjective nature of patient outcomes. The primary aim of this review is to provide a comprehensive analysis of the integration of digital workflows in prosthodontics, focusing on their impact on clinical outcomes, material advancements, and the challenges that hinder their broader adoption. By examining the latest literature, this review seeks to highlight the benefits and limitations of adopting digital technologies in prosthodontic practice while offering insights into their long-term potential in modern dental care.

## Review

Methodology

This review aims to evaluate the impact of integrating digital workflows into prosthodontic practice by conducting an extensive literature review. We searched relevant databases, including PubMed, Excerpta Medica database (EMBASE), Scopus, and Google Scholar, using search terms such as "digital workflow", "prosthodontics", "CAD/CAM", "intraoral scanning", "3D printing", and "digital prosthetics." The search was limited to studies published up to February 2024. We included literature from a variety of countries, which reflect a broad range of healthcare systems, from those with comprehensive dental insurance coverage to those with limited or no coverage for prosthodontic treatments. This international perspective is essential in understanding the diverse contexts in which digital workflows are being implemented.

The inclusion and exclusion criteria for this review are outlined in Table 1. Specifically, we focused on peer-reviewed studies that investigated the use of digital workflows in fixed prosthodontic treatments, including both tooth-borne and implant-supported prostheses. The studies evaluated key outcomes such as accuracy, time efficiency, cost-effectiveness, patient satisfaction, and clinical performance. The inclusion criteria permitted a wide range of study designs, while studies that involved analog methods, mixed digital and analog techniques, or were published in non-peer-reviewed sources were excluded.

**Table 1:** Inclusion and exclusion criteria CAD: computer-aided design; CAM: computer-aided manufacturing; IOS: intraoral scanners

Criteria	Inclusion	Exclusion
Publication type	Peer-reviewed studies in academic journals	Non-peer-reviewed sources or publications in non-academic outlets
Target population	Studies focusing on fixed prosthodontic treatments, including tooth-borne and implant-supported fixed cases	Studies unrelated to fixed prosthodontic treatments or focusing on disciplines outside of prosthodontics
Research focus	Studies examining the use of digital workflows (CAD/CAM, IOS, 3D printing) in fixed prosthodontic care	Studies not focused on the role of digital workflows in fixed prosthodontics or those involving analog methods
Study design	Randomized controlled trials, cohort studies, longitudinal, cross-sectional, and systematic reviews	Theoretical articles, commentaries, case reports, or studies without empirical data

Data extraction and synthesis

The data extraction process followed a narrative synthesis approach. Studies were grouped according to the specific digital technologies explored (e.g., CAD/CAM, IOS, 3D printing) and the clinical outcomes measured. The synthesis aimed to connect these technologies with prosthodontic outcomes such as accuracy, efficiency, and patient satisfaction, offering a comprehensive overview of the current evidence base on digital workflows in prosthodontics.

Review

Digital workflows have brought profound changes to prosthodontics, significantly enhancing the precision, time efficiency, and patient-centered outcomes of dental treatments. By integrating cutting-edge technologies such as IOS, CAD, and CAM, these workflows have transformed how dental prostheses are designed, produced, and delivered. This review offers a detailed examination of the most recent findings related to digital workflows, including accuracy, time efficiency, material advancements, patient satisfaction, and challenges to adoption.

Accuracy and Precision of Digital Workflows

The accuracy of prosthetic restorations is a critical factor in prosthodontics, and digital workflows have demonstrated significant improvements in this area. Siqueira et al. (2020) reported that fully guided implant surgeries using digital workflows achieved superior precision in implant placement compared to traditional workflows. This precision is especially important for implant-supported restorations, where minor errors can affect the long-term success of the prosthesis [5].

Joda and Brägger (2019) also found that CAD/CAM systems offer superior accuracy for both single and multi-unit restorations. Their five-year follow-up study demonstrated that digitally fabricated crowns provided more precise fits, significantly reducing the need for postoperative adjustments [2]. A systematic review by Bernauer et al. (2023) confirmed that digitally fabricated prostheses, particularly those made from monolithic zirconia, resulted in higher accuracy and fewer adjustments, enhancing the overall fit of the prosthesis [1].

In addition to implant-supported prostheses, significant advancements have also been made in the sector of tissue-supported prosthetics through digital workflows. Deng et al. (2023) compared digital and conventional workflows for complete denture fabrication, showing that digital methods reduced treatment time and improved fit and retention. The study also found that digital workflows led to fewer adjustments and improved patient satisfaction compared to conventional methods [8]. Similarly, Abdelnabi and Swelem (2024) reviewed clinical outcomes of 3D-printed dentures, noting improved clinical fit and patient-centered outcomes, such as comfort and satisfaction, compared to traditional dentures [9].

Hashemi et al. (2022) compared digital and conventional workflows, concluding that digital workflows offer greater precision, especially for complex restorations such as implant-supported prostheses. Their findings align with other studies emphasizing the accuracy and reliability of digital methods [10].

These findings, along with others comparing the accuracy and precision of digital versus conventional workflows, are summarized in Table 2.

**Table 2:** Accuracy of digital vs. conventional workflows CAD: computer-aided design; CAM: computer-aided manufacturing

Study	Prosthesis type	Workflow	Outcome
Siqueira et al. [5]	Implant-supported prostheses	Fully digital	Superior precision in implant placement
Joda and Brägger [2]	Single/multi-unit prostheses	CAD/CAM	More accurate than conventional methods
Bernauer et al. [1]	Tooth-borne and implant-supported prostheses	Full digital	Fewer post-operative adjustments
Deng et al. [8]	Tissue-supported prostheses	Full digital	Reduced treatment time, better fit, and retention
Abdelnabi and Swelem [9]	Tissue-supported prostheses	Full digital	Improved clinical fit, greater patient comfort
Hashemi et al. [10]	Complex implant prostheses	Full digital	Higher precision, fewer errors

Material Advancements in Digital Prosthodontics

The rise of digital workflows has been accompanied by the development of advanced materials for prosthetic restorations. Monolithic zirconia and lithium disilicate are now widely used due to their strength, durability, and esthetic properties, making them ideal for use in digital workflows.

Blatz & Conejo (2019) reported that monolithic zirconia is particularly well-suited for posterior restorations due to its fracture resistance and high strength. Lithium disilicate, on the other hand, is preferred for anterior restorations because of its esthetic translucency [3].

Michelinakis et al. (2021) also noted that 3D printing offers additional flexibility for fabricating complex prosthetic designs, particularly for custom temporaries and surgical guides [11]. Siqueira et al. (2020) similarly highlighted the use of 3D-printed resins in complex implant-supported prostheses, citing their cost-effectiveness and ease of fabrication [6, 5].

These advancements in materials, including monolithic zirconia, lithium disilicate, and 3D printed resins, are detailed in Table 3, highlighting their specific properties and applications in digital prosthodontics.

**Table 3:** Digital prosthodontic materials

Material	Properties	Study
Monolithic zirconia	High strength, wear resistance	Blatz and Conejo [3]
Lithium disilicate	Excellent esthetics, ideal for anterior teeth	Joda and Brägger [2]
3D-printed resins	Customizability, cost-effective temporaries	Michelinakis et al. [11]; Siqueira et al. [5]

Patient Satisfaction and Clinical Outcomes

Patient satisfaction is a critical outcome in prosthodontics, and digital workflows have been shown to significantly enhance patient experiences. Joda and Brägger (2019) found that patients receiving digitally fabricated prostheses reported higher levels of satisfaction due to reduced discomfort during the impression process and the improved precision of the final restorations [2].

Siqueira et al. (2020) also observed that patients undergoing implant treatments with fully digital workflows experienced fewer postoperative adjustments and were generally more satisfied with the esthetic outcomes of their prostheses compared to those treated with traditional workflows [5].

D’Ambrosio et al. (2023) conducted an umbrella review comparing digital and conventional dental impressions and found that patients overwhelmingly preferred digital workflows. Patients reported greater comfort during the impression process, reduced gagging, and a faster overall experience, which significantly contributed to their satisfaction with digital prosthetic treatments [12].

These findings regarding patient satisfaction with digital workflows, including increased comfort, fewer adjustments, and improved clinical outcomes, are summarized in Table 4.

**Table 4:** Patient satisfaction with digital workflows

Study	Prosthesis Type	Outcome
Joda and Brägger [2]	Tooth-borne prostheses	Higher satisfaction due to comfort and precision
Siqueira et al. [5]	Implant-supported prostheses	Increased comfort, fewer adjustments
D’Ambrosio et al. [12]	Digital implant prostheses	Higher patient satisfaction with digital impressions

Challenges and Barriers to Adoption 

Despite the many advantages of digital workflows, significant barriers remain to their widespread adoption. The high initial cost of acquiring digital equipment such as intraoral scanners, CAD/CAM systems, and 3D printers is a major challenge. Mishra and Chowdhary (2021) highlighted that the financial burden of purchasing and maintaining digital equipment can be prohibitive, particularly for smaller practices [7].

In addition to financial challenges, the learning curve associated with mastering digital workflows can also deter clinicians from transitioning from traditional methods. Agnini et al. (2015) emphasized that ongoing training and familiarity with digital systems are essential for clinicians to fully benefit from the accuracy and efficiency offered by these technologies [13].

Moreover, the need for software and hardware updates can add to the cost and complexity of maintaining a digital workflow. Michelinakis et al. (2021) noted that regular updates are necessary to ensure that digital systems remain compatible with the latest materials and fabrication techniques [6]. Table 5 outlines key barriers to adopting digital workflows, including costs, training, and software updates.

**Table 5:** Barriers to adoption of digital workflows

Barrier	Description	Study
Initial costs	High cost of equipment and software	Mishra and Chowdhary [7]
Training requirements	Steep learning curve for clinicians	Angini et al. [13]
Software updates	Regular updates required for optimization	Michelinakis et al. [6]

Limitations

This review has several limitations that should be acknowledged. First, it primarily focuses on studies published in English and within the last decade, which may exclude earlier or non-English research that could provide valuable insights. Additionally, much of the literature emphasizes short- to mid-term outcomes, with limited data available on the long-term success and durability of digital workflows, particularly in complex, multi-unit restorations. The variability in study designs and outcomes, as well as differences in the technologies and materials used across studies, makes it difficult to draw definitive conclusions about the superiority of digital workflows in all clinical situations. Moreover, the review is reliant on the quality of the included studies, some of which may have inherent biases, particularly those conducted in controlled, institutional settings rather than real-world dental practices.

## Conclusions

Digital workflows have brought transformative changes to prosthodontics, improving accuracy, efficiency, and patient satisfaction. By utilizing technologies such as intraoral scanners, CAD/CAM systems, and 3D printing, clinicians can deliver highly precise prosthetic restorations in less time and with fewer postoperative adjustments. Despite the significant barriers to adoption, including high initial costs and the need for ongoing training, digital workflows are poised to become a mainstay in prosthodontic practice as technology advances and becomes more accessible.
